# An invasive phenotype induced by relaxation of DNA supercoiling in Campylobacter jejuni triggers disruption of tight junctions of intestinal spheroids

**DOI:** 10.1099/mic.0.001560

**Published:** 2025-06-05

**Authors:** Matthew V.X. Whelan, Laura Ardill, Jeremy C. Simpson, Tadhg Ó Cróinín

**Affiliations:** 1 School of Biomolecular and Biomedical Science, University College Dublin, Belfield, Dublin 4, Ireland; 2 School of Biology and Environmental Science, University College Dublin, Belfield, Dublin 4, Ireland

**Keywords:** *Campylobacter*, cytoskeleton, invasion

## Abstract

The relaxation of DNA supercoiling in *Campylobacter jejuni* leads to increased protein secretion and a more invasive phenotype, but little is known about the specific mechanisms involved. The aim of this study was to elucidate how these induced bacteria interact with epithelial cells to mediate invasion using different cell models. In HT29 epithelial cell monolayers, pre-treatment of *C. jejuni* with novobiocin to relax DNA supercoiling significantly increased bacterial association and invasion, forming clusters at cell junctions. This invasive phenotype, which we term *C. jejuni* supercoiling induced (SI), led to marked disruption of tight junctions (TJs) and adherens junctions, as evidenced by the loss of occludin and *β*-catenin signal during infection. In a 3D spheroid model, *C. jejuni* (SI) displayed increased association with and penetration into the centre of spheroids, although significant disruption of their integrity was not observed. Further investigation revealed that cytoskeletal dynamics play a pivotal role in this process; inhibition of microtubule polymerization, but not actin polymerization, rescued the *β*-catenin disruption induced by *C. jejuni* (SI), highlighting microtubules as key targets for *C. jejuni* virulence. This study reveals that SI invasion by *C. jejuni* is associated with the disruption of TJs, suggesting a paracellular route of invasion.

## Introduction

*Campylobacter jejuni* is a bacterial gastrointestinal pathogen that typically exists as a non-invasive microaerophilic commensal in the intestinal tract of avians such as broiler chickens [1]. However, its ability to survive environmental challenges allows for food-borne transmission to the human intestine, where it shifts to a highly infectious phenotype and causes severe gastroenteritis and, rarely, other complications such as Guillain–Barrè syndrome [2]. The mechanisms behind the transition from commensal to virulence phenotype of *C. jejuni* are not well understood, but surface factors such as adhesion and capsid proteins, lipooligosaccharide [3] and flagella [4] have been implicated. Secreted factors such as outer membrane vesicles [5], cytolethal distending toxins [6] and the *Campylobacter* invasion antigen (Cia) family [7] have also been reported to play roles in promoting invasion and facilitating intracellular survival.

Uncovering key bacterial determinants of infection and the kinetics of host-cell disruption can be challenging due to the lack of a tractable model to study the regulation of virulence factors. Our research has previously shown that growth in subinhibitory levels of novobiocin reduces the activity of GyrB and leads to the relaxation of negative DNA supercoiling and induction of an invasive phenotype in a poorly invasive strain [8] and that chicken mucous modulates *C. jejuni* supercoiling homeostasis [9]. This ability to specifically and dose dependently increase invasiveness through DNA relaxation using novobiocin offers a novel model for understanding the host cell mechanisms involved in virulence. We also found that the relaxation of DNA supercoiling associated with the acquisition of fluoroquinolone resistance through a single-point mutation increases virulence in the *Galleria mellonella* model and increases the viability of biofilms under aerobic conditions [10,11].

The debate over whether *C. jejuni* utilizes transcellular or paracellular transport from the intestinal lumen to target enterocytes has been ongoing, with evidence for both [1,12]. The extracellular matrix protein fibronectin is a key interactor for *C. jejuni* adhesins and receptor for bacterial uptake, and its localization predominantly on the basolateral membrane of enterocytes supports a paracellular route [13,14]. The paracellular space is kept tightly closed by tight junctions (TJs) and adherens junctions (AJs), and for a bacterial pathogen to penetrate an epithelial monolayer through this space, it must have mechanisms to disrupt these structures. TJs are apical cell-cell attachments that promote differentiation of the plasma membrane into apical (mucosal) and basolateral (serosal) regions and restrict transport [15,16]. They are a key barrier to *C. jejuni* invasion of the intestinal epithelium [17]. *C. jejuni* has been found to disrupt TJ integrity 24 h post-infection (hpi), but the mechanisms by which it does so are not well understood [18]. Recent studies have suggested that the release of outer membrane vesicles enriched for serine proteases can disrupt both TJs and AJs and promote invasion of intestinal epithelial cells [5,19, 20]. AJs are cell-cell adhesion structures that *C. jejuni* must bypass during paracellular translocation [21]. The core component of AJ is E-cadherin, a transmembrane protein that forms homodimers with neighbouring cells and interacts with actin microfilaments for support. Studies have suggested that bacterial serine proteases play a role in degrading AJ by *C. jejuni*. The serine protease HtrA, for example, selectively cleaves the NTF domain of E-cadherin during infection, and outer membrane vesicles enriched for HtrA, cj0511 and cj1365c have been shown to mediate degradation of T84 cell E-cadherin [22,24].

In 2007, researchers found that Rac1 and Cdc42 were activated during *C. jejuni* infection of intestinal epithelial cells, indicating a key role for remodelling of the cortical actin cytoskeleton during invasion [25]. The literature previously reported conflicting evidence on whether actin microfilaments or microtubules were implicated in *C. jejuni*-mediated invasion [26]. However, it has been suggested that the role of actin polymerization is more important than that of microtubules. This is supported by studies that show treatment with inhibitors of actin polymerization had similar effects to treatment with both actin and microtubule inhibitors [17,27] and that Rho GTPases play a key role in membrane ruffling induced by *C. jejuni* invasion [27]. The virulence effector CiaC plays a role in recruiting Rac1 during *C. jejuni* invasion, but the mechanism is not yet understood [28].

Developing 3D intestinal models for infections requires consideration of the apical/basolateral orientation of the cells. For example, enteroid cysts naturally polarize with the basolateral membrane facing the external surface, requiring microinjection of pathogens into the cyst lumen to model infection. There have been limited 3D cell culture studies of *C. jejuni* infections, but some promising work on *Salmonella* Typhimurium infection of 3D spheroids has been done. In 2006, Höner *et al.* adapted a rotating wall vessel-derived intestinal spheroid model using colonic HT29 cells for infection with *S*. Typhimurium [29]. They found that when grown in 3D, the HT29 cells presented with a physiological distribution of TJ and AJ proteins, indicating highly differentiated cells. *S*. Typhimurium infection of the spheroids caused physiological and cell surface structural changes, and *S*. Typhimurium lacking the SPI-I T3SS could invade the spheroids but did not induce physiological changes. The data suggests that using highly differentiated spheroid models can provide a better understanding of host-pathogen interactions.

In light of this, the goal in this study was the characterization of the previously described more invasive phenotype in *C. jejuni* associated with the relaxation of DNA supercoiling and specifically to investigate effects on cytoskeletal remodelling, internalization and interaction with cell-cell structures in intestinal epithelial cell lines and an intestinal spheroid model.

## Methods

### Bacterial strains and growth conditions

All strains used in this study were stored as stock solutions containing 80% Mueller–Hinton (MH) broth and 20% glycerol at −80 °C. *C. jejuni* strains were grown at 37 °C for 48 h under microaerophilic conditions, and *S.* Typhimurium strain SL1344 was grown under normal atmospheric conditions. To revive the stocks, bacteria were streaked on MH agar and incubated for 48 h. Liquid cultures were grown at a starting OD_600_ of 0.02 under the same conditions.

### Treatment of strains with novobiocin to relax DNA supercoiling

The relaxation of negative DNA supercoiling was done as previously described, using novobiocin at subinhibitory concentrations to dose dependently inhibit the DNA gyrase B subunit [8]. Strains were grown in the presence of 5, 10 or 15 µg ml^−1^ novobiocin for 18 h at 37 °C at 200 r.p.m. shaking. Labels ‘*C. jejuni* supercoiling induced (SI)’ indicate NCTC11168 pre-incubated with 10 µg ml^−1^ novobiocin. All bacterial suspensions were supplemented with appropriate concentrations of novobiocin to maintain supercoiling states for subsequent experiments. Growth of *C. jejuni* in the presence of 10 µg ml^−1^ of novobiocin has previously been shown to relax DNA supercoiling and does not affect the growth rate of *C. jejuni* [9]. Treatment of HT-29 cells with 10 µg ml^−1^ novobiocin had no effect on TJs or cell viability as outlined in Fig. S1 (available in the online Supplementary Material), showing that the effect was mediated directly through altering the DNA supercoiling of the bacteria.

### Viability staining of bacteria

To assess viable invasion, *C. jejuni* and *S*. Typhimurium strains were labelled with 5-TAMRA-SE, a cell-permeant dye that is crosslinked by bacterial esterases upon entering the cytosol, providing a bright indicator of live bacteria. Overnight cultures were equalized to an OD_600_ of 0.2, centrifuged and resuspended in PBS containing 40 µg ml^−1^ TAMRA and incubated for 30 min under microaerophilic or aerobic conditions, respectively. The labelled strains were washed (4×) in PBS and resuspended in MH broth before being used in the infection assay.

### Growth and infection of HT29 monolayers

Fifty thousand HT29 cells/cm^2^ were seeded into 12-well plates (Corning) containing 16 mm poly-lysine-coated optical coverslips (VWR) or into CellCarrier-96 Ultra 96-well plates (PerkinElmer), for 3 days (for cell island) and 7, 10 or 14 days (monolayer), changing the media every 2 days. Bacterial strains were grown on MH agar for 2 days in microaerophilic conditions and then equalized at an OD_600_ of 0.02 in MH broth and incubated for 18 h at 37 °C, shaking at 200 r.p.m. under microaerophilic conditions. To obtain an m.o.i. of 100 : 1, the bacteria were then centrifuged at 4,000 r.p.m. for 10 min and equalized to an OD_600_ of 0.2 in McCoy’s 5A media supplemented with 10% heat-inactivated FBS. The HT29 monolayer was washed once with 37 °C PBS, and the media were replaced with McCoy’s 5A infected with an OD_600_ of 0.2 of *C. jejuni* or * S*. Typhimurium. The bacteria were incubated with the HT29 monolayer for 3 h under microaerobic conditions. The wells were then washed 3× with PBS, and the cells were fixed for 15 min with 4% paraformaldehyde for 10 min in ice-cold methanol.

### Growth and infection of polarized HT29 monolayers

For infection assays on polarized epithelial monolayers, 5,000 HT29 cells/cm^2^ were seeded into transwell inserts with a pore size of 0.4 µm. The cells were incubated for 30 days to allow for differentiation of the monolayer. *C. jejuni*, *C. jejuni* induced by preincubation with 10 µg ml^−1^ novobiocin [*C. jejuni* (SI)] and *S*. Typhimurium were added onto the polarized monolayers for 6 and 24 h in ideal gas conditions.

### Growth of HT29 spheroids in 3D Matrigel matrix

A 3D cell culture approach based on a matrix-based overlay method consists of seeding HT29 cells on top of a thin layer of Matrigel dispensed in wells of optically clear multiwell plates [30]. The matrix-based overlay method has been reported to facilitate the assembly of large numbers of non-uniform spheroids in wells, being suitable for *in situ* Immunfluorescence processing and imaging of 3D structures [30,32]. To grow spheroids for image-based high-content imaging, the wells of PhenoPlate 96-well microplates (Revvity) were manually coated with 15 µl of Matrigel (Corning) at a final 4 mg ml^−1^ concentration (in serum-free McCoy’s 5A medium). The 96-well plates were centrifuged at 4 °C at 900 r.p.m. for 15 min. This step was then followed by incubation for 30 min at 37 °C to allow gelation of the matrix in wells. After this settlement of the Matrigel bed, HT29 cells (3,000 cells per well) were dispensed in complete McCoy’s 5A medium (without phenol red) to a final volume of 50 µl. After one h of incubation at 37 °C, the overlay was supplemented with 10 µl of complete medium containing the required amount of Matrigel to give a 2% (w/v) final concentration in 60 µl volume. On the second day of 3D culture, the complete growth medium was changed. Spheroids were allowed to grow for 4 days of 3D cell culture.

### Treatment of HT29 spheroids with cytoskeleton inhibitors

To facilitate the disassembly of microtubules, spheroids were treated with a complete medium containing 33 µM nocodazole (Sigma) for 3 h. Bacterial infection was conducted in the presence of a low concentration of nocodazole (3 µM) for the duration of invasion (24 or 6 h). Cytochalasin D: To perturb the actin cytoskeleton, spheroids were treated with a complete medium containing 10 µM cytochalasin D (ThermoFisher) for 90 min. Bacterial infection was conducted in the presence of a low concentration of cytochalasin D (1 µM) for the duration of invasion (48, 24 or 3 h).

### Total association and gentamicin protection assays

For quantifying the total association and internalization of *C. jejuni* after 3 h of invasion on HT29 cell monolayers, invasion assays were performed as previously described. Additionally, the c.f.u. of total associated bacteria and bacteria internalized into the intracellular compartment were quantified via the gentamicin protection assay. To quantify the total association after 3 h of invasion, the cells were washed three times with PBS and lysed with 1% saponin in PBS for 15 min. Serial dilutions of the cell lysates were done using MH broth and plated on MH agar. The plates were incubated for 48 h under microaerophilic conditions at 37 °C, followed by counting of c.f.u. To quantify internalized bacteria after 3 h of invasion, the infected HT29 cells were incubated for an additional 2 h in complete McCoy’s 5A medium supplemented with 200 µg ml^−1^ gentamicin sulphate (Lonza), then washed three times with PBS and lysed, followed by colony quantification.

### Autoagglutination assay

Overnight MH broth cultures of *C. jejuni* and *C. jejuni* grown in MH broth supplemented with 5, 10 and 15 µg ml^−1^ of novobiocin were equalized to an OD_600_ of 0.6 and incubated statically at room temperature RT) for 0, 3 and 24 h. One millilitre of the top fraction of the broth was removed at the timepoints, and the OD_600_ was determined.

### Infection of HT29 spheroids

The bacteria used for invasion assays of healthy HT29 spheroids were *C. jejuni*, *C. jejuni* (induced) with 10 µg ml^−1^ novobiocin [*C. jejuni* (SI)] and *S*. Typhimurium. The bacteria chosen for invasion of actin- and tubulin-inhibited HT29 spheroids (nocodazole and cytochalasin D treated) were *C. jejuni* (SI) and *S*. Typhimurium. To obtain an m.o.i. of 100 : 1, the bacterial strains were then centrifuged at 4,000 r.p.m. for 10 min and equalized in McCoy’s 5A media (without phenol red) supplemented with 10% heat-inactivated FBS in a final volume of 50 µl. The HT29 spheroids were washed once with PBS at 37 °C, and the medium was replaced with McCoy’s 5A medium infected with *C. jejuni* or *S*. Typhimurium. The bacteria were incubated with the HT29 spheroids for 4 h or 24 h under ideal gas conditions for the HT29 cells.

### Immunofluorescence staining of HT29 monolayers and 3D spheroids post-invasion

After each infection timepoint was reached, the monolayers and spheroids were processed for immunofluorescence following a protocol established in 2D monolayers [33]. The wells were washed once with PBS (pH 7.4) prior to fixation with 4% (w/v) PFA solution for 30 min at RT. All processing steps were performed at 20 °C. Permeabilization of cellular membranes was carried out with 0.5% saponin/0.1% Triton TX100 in PBS containing 0.5 % BSA and 50 mM NH4Cl for 1 h, followed by three washes with PBS. Then, the spheroids were incubated for 2 h with a 1° antibody targeting mouse anti-paxillin, mouse anti-occludin or mouse anti-*β*-catenin in blocking, followed by three times washes with PBS. Thirdly, the monolayers/spheroids were incubated with a 2° antibody of goat anti-mouse-Alexa488 (to stain occludin/*β*-catenin) in blocking solution that also contained 200 µg µl^−1^ Hoechst33342 (nuclei, blue) and 2 units ml^−1^ of Phalloidin Alexa647 (actin, cyan) to counterstain nuclei and the actin cytoskeleton, respectively. Finally, the monolayers/spheroids were washed with PBS three times and kept in PBS for subsequent automated acquisition of images.

### Fluorescence microscopy

Confocal images were acquired using the FluoView FV1000 laser scanning microscope (Olympus) with a 60×/1.35 NA UPlanSApo oil immersion objective. Confocal Z-stack acquisition was acquired at 1 µm intervals. Maximum Z-projections and volumetric reconstructions of confocal Z-stacks were constructed using Fiji ImageJ stack processor and ‘volume viewer’ plugin. All confocal images of HT29 monolayers were processed using Fiji ImageJ [34]. Automated confocal microscopy was carried out using an Opera Phenix (Revvity) high-content screening microscope using a 20×/1.0 NA water objective. The resulting images had an effective xy resolution of 0.66 µm. For each condition and timepoint, 25 sub-positions were acquired over two replicates, possessing four colour channels: blue (405 nm), green (488 nm), near-red (561 nm) and far-red (cyan, 640 nm). For each sub-position, 50 Z-slices were acquired at 1 µm intervals to allow for complete reconstruction of the 3D spheroid for quantification.

### Image analysis

Image analysis of confocal Z-projections was carried out using Fiji ImageJ and CellProfiler [34,35]. The analysis pipeline is shown in the supplementary information. A high-throughput image analysis approach was developed using the Columbus image data and analysis system v.2.8.2 (PerkinElmer). This analysis pipeline titled ‘Mothra’ was constructed based on an equatorial plane-based analysis pipeline ‘Godzilla’ published previously [30]. The building blocks involved in the analysis pipeline are shown in Table S1.

### Statistical analysis

**Fig. 1.**
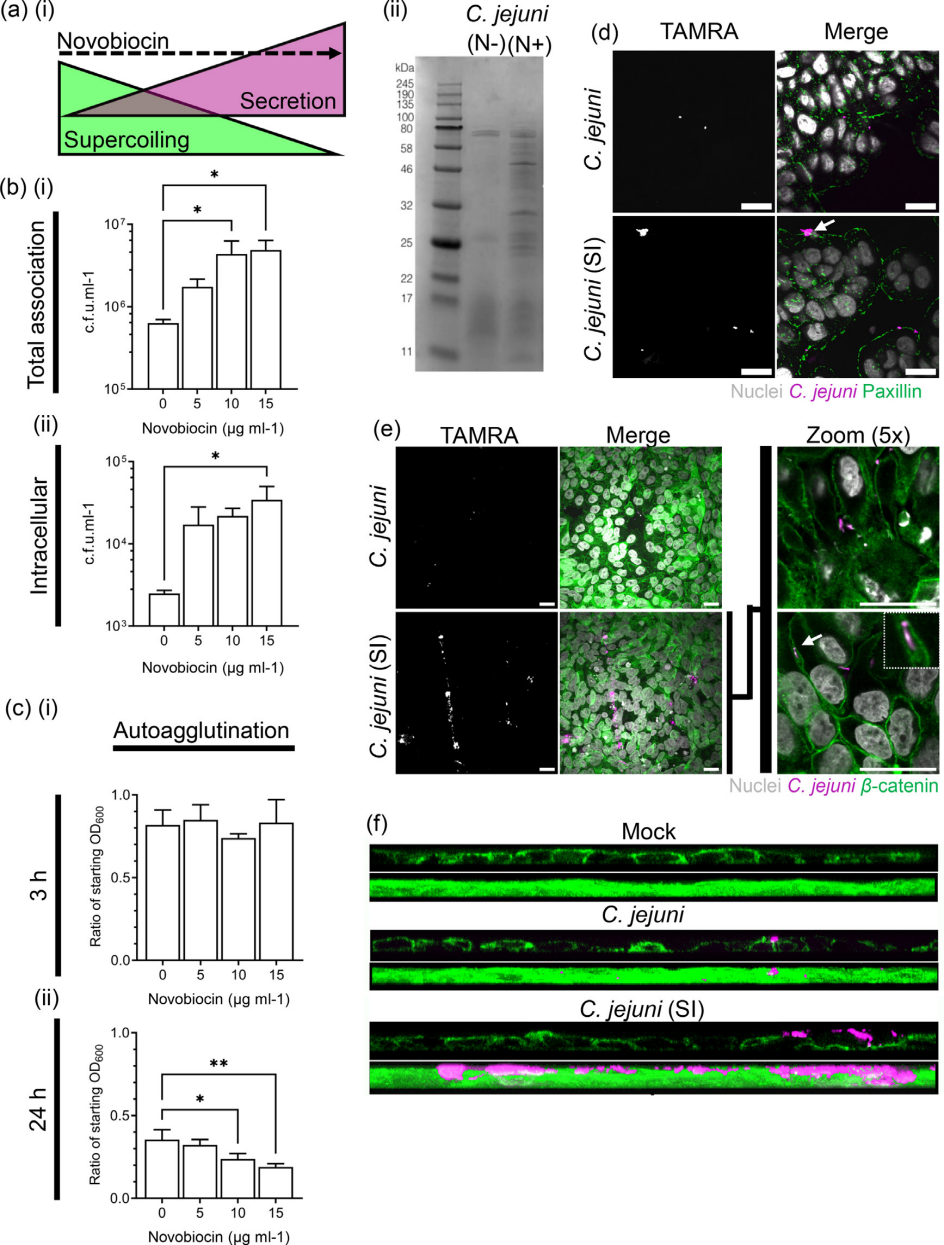
Relaxation of DNA supercoiling promotes an invasive phenotype in *C. jejuni*. DNA supercoiling can be dose dependently relaxed by treatment with subinhibitory levels of novobiocin, which leads to increased secretion (**a, i**) as confirmed by SDS electrophoresis for *C. jejuni* grown in the presence of 10 µg ml^−1^ novobiocin as compared to those grown without. (**b**) Preincubation with novobiocin leads to a log increase in cellular (**i**) total association and (ii) intracellular *C. jejuni* at 3 hpi (*N*=3). (**c**) Autoagglutination assays measuring reductions in OD_600_ over time due to autoagglutination show that bacterial clumping does not play a role at 3 hpi with a slight effect at 24 h. (**d**) A novobiocin-induced invasive phenotype [*C. jejuni* (SI), magenta] preferentially associates infection clusters of bacteria to epithelial integrins as seen by association with paxillin (green) at 1 hpi. (**e**) *C. jejuni* (SI) clusters form preferentially at *β*-catenin (green) positive cell-cell borders and preferentially translocate paracellularly (white arrow). Scale=20 µm. (**f**) 3D reconstruction XZ-profile of *β*-catenin monolayer shows preferential localisation of *C. jejuni* (magenta) to the cell-cell borders and demonstrates specific infiltration of the *C. jejuni* (SI) into the monolayer and localized associated loss of *β*-catenin intensity. Scale=20 µm

**Fig. 2.**
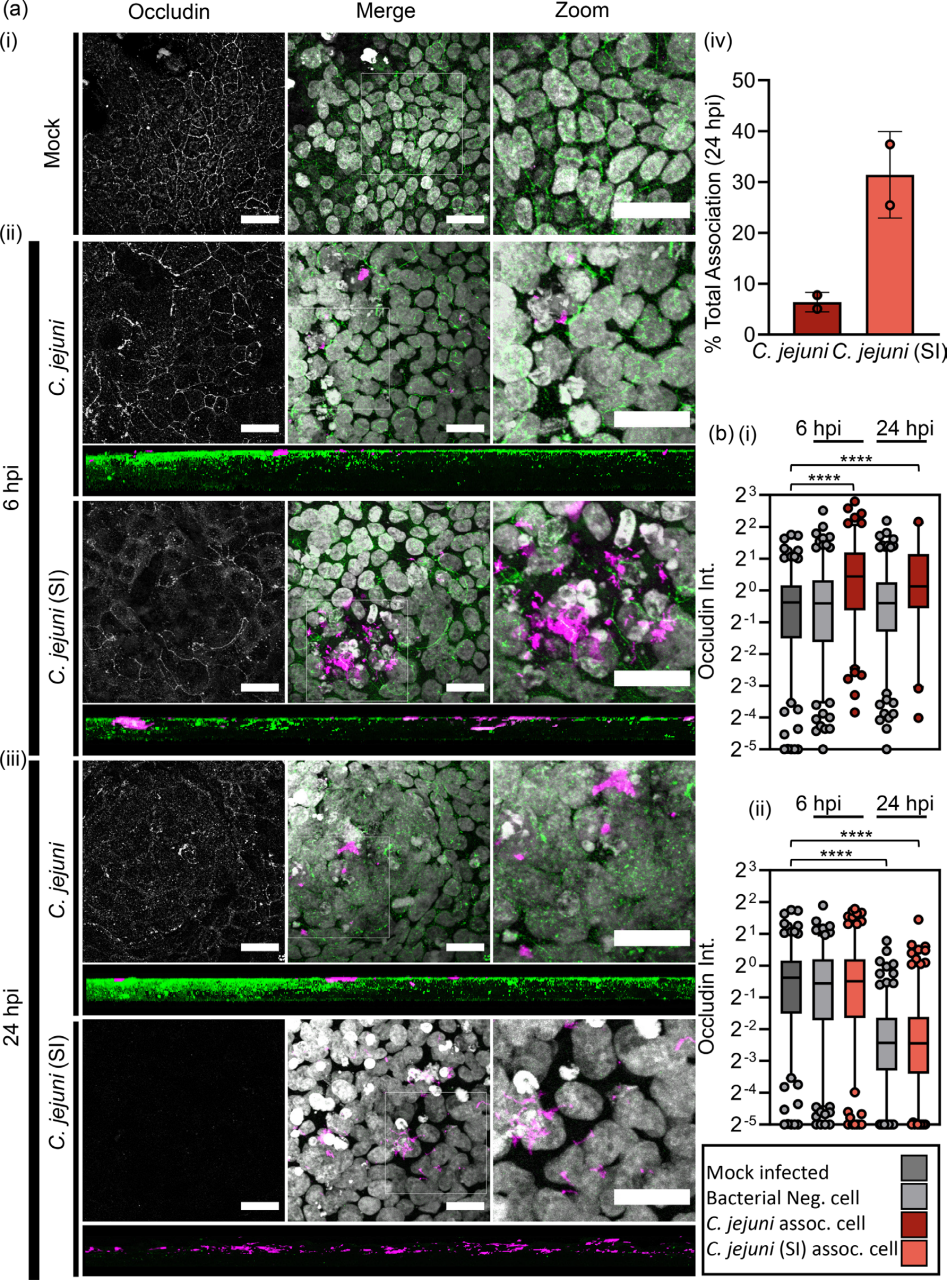
Induced invasive phenotype enhances kinetics of TJ disruption. (a, i–iii) Confocal Z-stack and XZ 3D reconstruction of polarized (**i**) HT29 (blank) and HT29 monolayers inoculated with *C. jejuni* and *C. jejuni* (SI) at (ii) 6 hpi and (iii) 24 hpi highlights *C. jejuni* (SI) disruption of occludin intensity. Bacteria were immunofluorescently labelled with TAMRA (magenta). The HT29 monolayer was labelled for host cell TJs (anti-occludin-A488, green) and nuclei (Hoechst33342, white). *N*=2. Scale=20 µm. (iv) Quantification of the total association at 24 hpi. *N*=2. (**b**) Single-cell quantification of occludin intensity of bacteria negative and positive cells at 6 and 24 hpi.

**Fig. 3.**
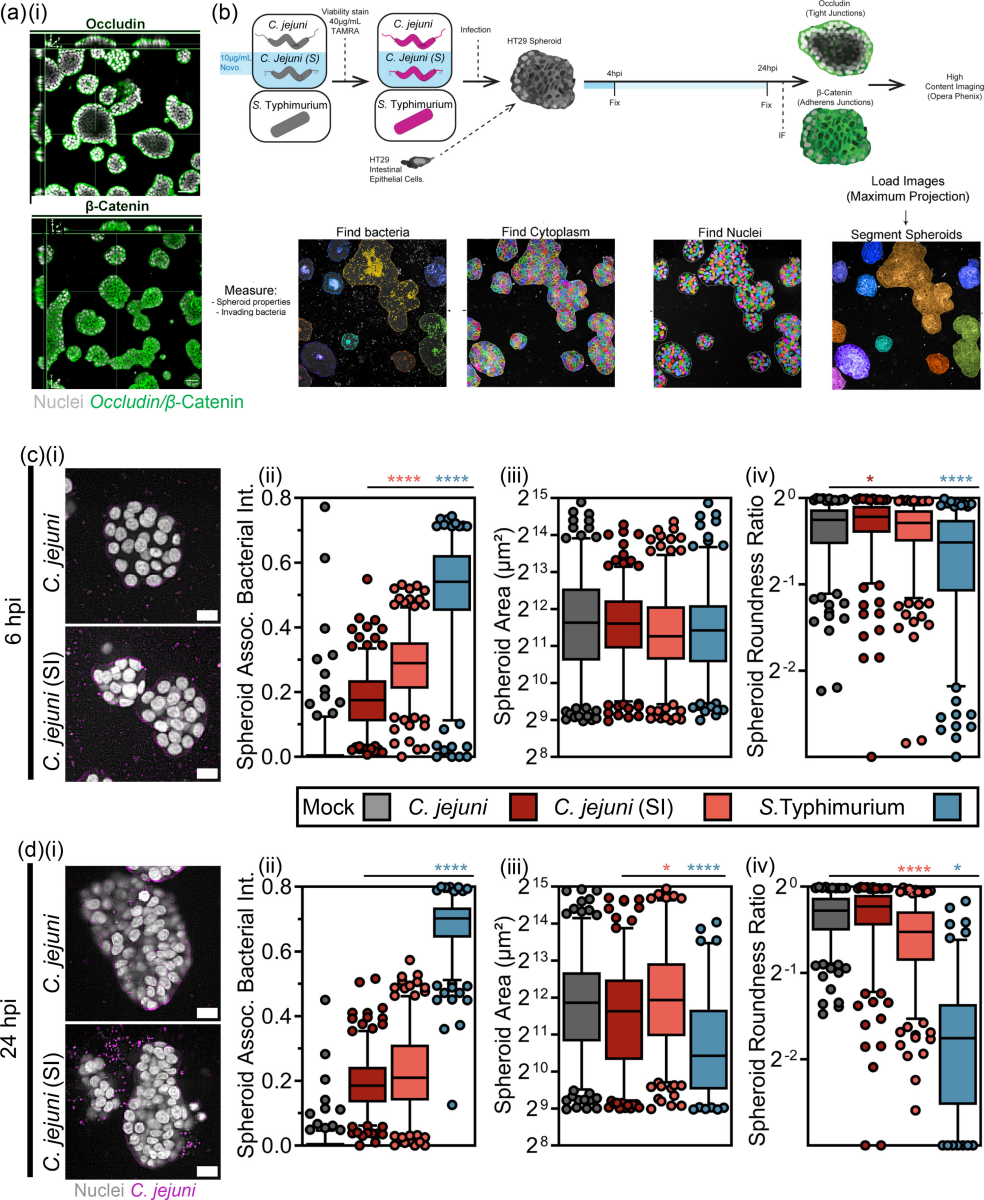
*C. jejuni* (SI) phenotype displays increased invasion and morphological disruption of intestinal spheroids. (**a**) XYZ maximum projection of 4-day-old HT29 spheroids immunofluorescently labelled with Hoechst33342 (nuclei, white), anti-*β*-catenin-A568 (AJs, green) or anti-occludin-A568 (TJs, green). Spheroid polarization is evident. (**b**) Infographic of Columbus image analysis workflow for 3D screen of HT29 spheroids. Medial slice of spheroids infected with *C. jejuni* and *C. jejuni* (SI) (magenta) at 6 hpi (**i**) and 24 hpi. Scale=20 µm. Single-spheroid quantification measuring mean segmented bacteria per spheroid at 6 hpi (c, ii) and 24 hpi (**d, ii**). Spheroid area at 6 hpi (c, iii) and 24 hpi (d, iii) and spheroid roundness ratio at 6 hpi (**c, iv**) and 24 hpi (**d, iv**). Each (•) represents a spheroid. (**b**) Data shown is mean±sd of 300 spheroids per condition. *N*=2. *S.* Typhimurium showed increased associated bacteria.

**Fig. 4.**
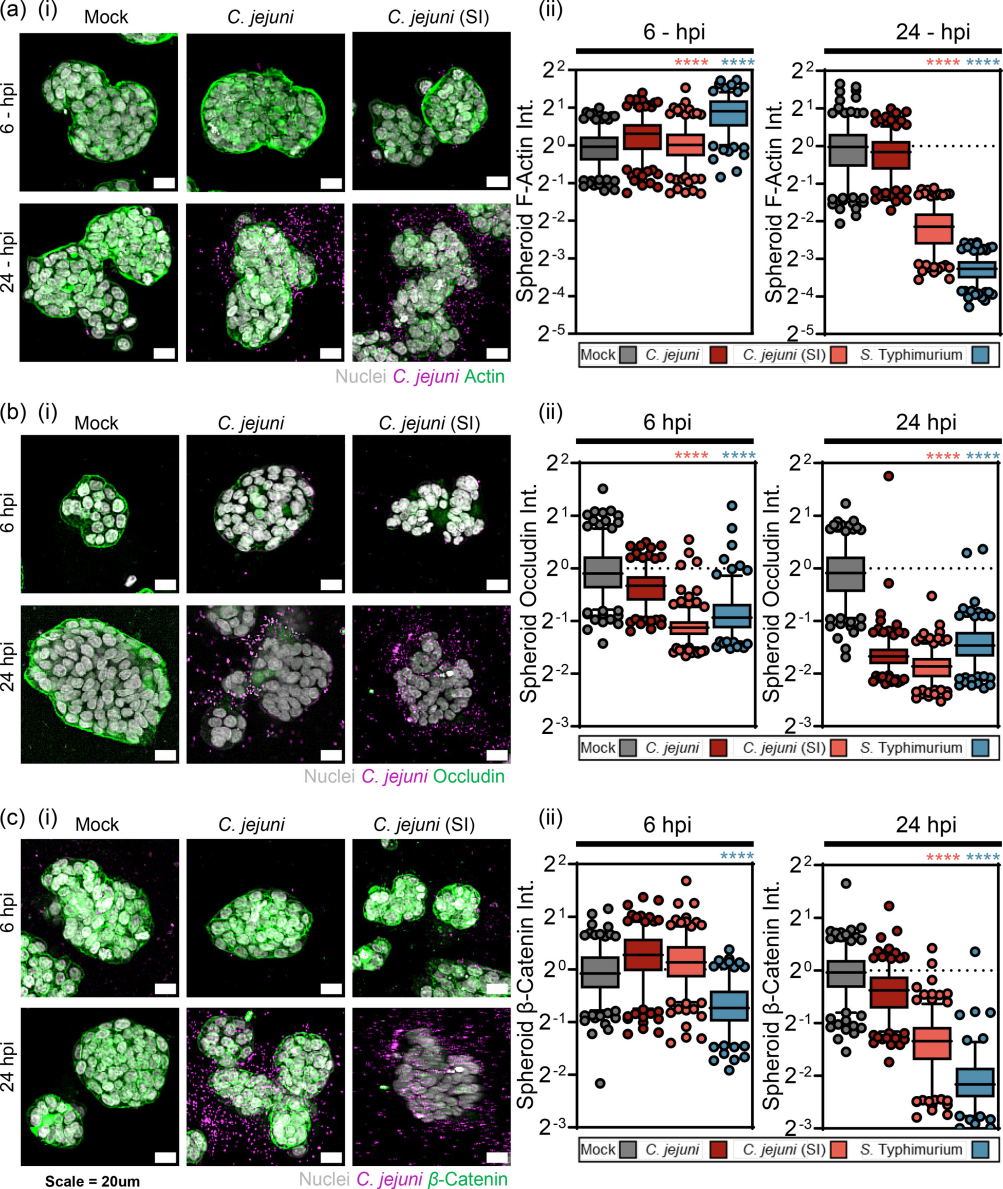
*C. jejuni* (SI) phenotype disrupts TJs during early infection and AJs in later infection. (**a, i**) Quantification of actin (green) intensity of healthy spheroids after (ii) 6- and 24-h incubation with control media (blank), *C. jejuni*, *C. jejuni* (SI) (induced with 10 µg ml^−1^ novobiocin) or *S.* Typhimurium SL1344 (bacteria, magenta). (**b, i**) Quantification of occludin (green) intensity of healthy spheroids after (ii) 6 h and 24 h incubation with control media (blank), *C. jejuni*, *C. jejuni* (SI) (induced with 10 µg ml^−1^ novobiocin) or *S.* Typhimurium SL1344 (bacteria, magenta). (**c, i**) Quantification of *β*-catenin (green) intensity of healthy spheroids after (ii) 6- and 24-h incubation with control media (blank), *C. jejuni*, *C. jejuni* (SI) (induced with 10 μg ml^−1^ novobiocin) or *S*. Typhimurium SL1344 (bacteria, magenta). Quantification is mean intensity (A.U.) per spheroid. Each (•) represents a spheroid. (**b**) Data shown is mean±sd of 250 spheroids per condition. *N*=2

All data quantitated was subjected to a Shapiro–Wilk normality test. Normally distributed data in Fig. 1 was subjected to an ordinary one-way ANOVA. All single-cell and single-spheroid intensity metrics (Figs2, 3, 4  5) were normalized to the median intensity value of the respective uninfected population. All single-cell data was subjected to a Kruskal–Wallis statistical test with a Dunn’s multiple comparison post hoc test. Statistical significance *P* value is represented as follows: 0.1234 (ns), 0.0332 (*), 0.0021 (**), 0.0002 (***) and <0.0001 (****).

**Fig. 5.**
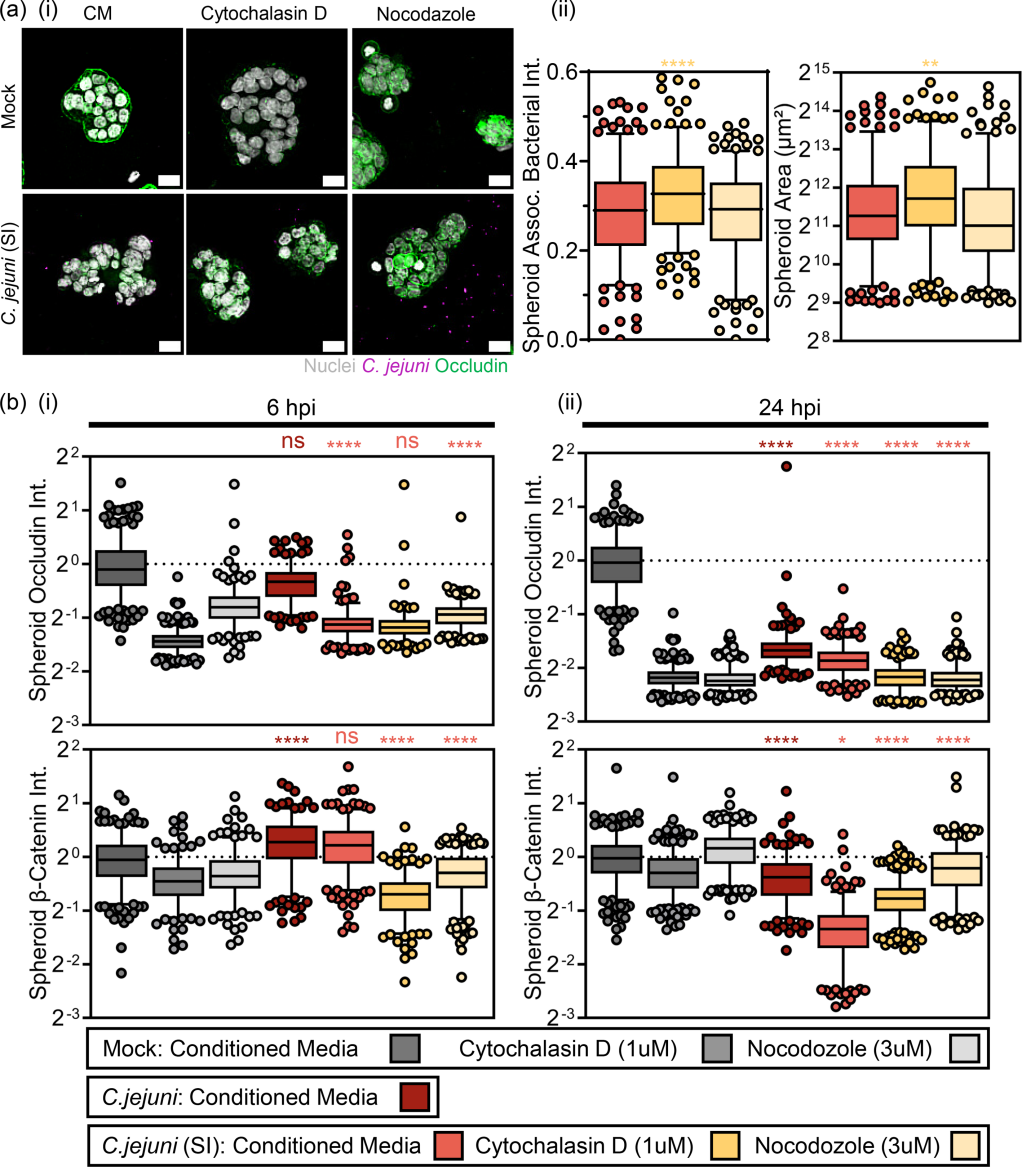
*C. jejuni* (SI) phenotype triggers disruption of TJs and AJs require microtubule dynamics. (**a, i**) Maximum projection of 4-day-old HT29 spheroids preincubated with 33 µM nocodazole (maintained at 3 µM during infection), or 10 µM cytochalasin D (maintained at 1 µM during infection) mock infected or infected with *C. jejuni* (SI) (magenta) labelled with Hoechst33342 (nuclei, white) and anti-occludin-A568 (TJs, green). Scale=20 µm. (**a, ii**) Single-spheroid quantification measuring mean segmented bacteria per spheroid (left) and spheroid area (right). (**b**) Quantification of occludin and *β*-catenin intensity of healthy spheroids vs cytochalasin D- and nocodazole-treated spheroids after (**i**) 6-h and (ii) 24-h infection with *C. jejuni* (SI) (induced with 10 µg ml^−1^ novobiocin). Quantification is mean actin intensity (A.U.) per spheroid. Each (•) represents a spheroid. Data shown is mean±sd of 250 spheroids per condition.

## Results

### Relaxation of negative DNA supercoiling promotes an invasive phenotype in *C. jejuni*

The relaxation of DNA supercoiling with subinhibitory concentrations of novobiocin has previously been shown to result in increased secretion, and this relationship was confirmed, showing an increase in secretion associated with treatment with novobiocin at 10 µg ml^−1^ [Fig. 1a(i, ii)]. To prove that these treated bacteria were more invasive in the HT29 intestinal epithelial cells used in this study, the *C. jejuni* type strain NCTC11168 was incubated for 18 h in MH broth or MH broth supplemented with a range of novobiocin concentration to pre-relax the bacterial chromosome. Bacteria were then labelled with the viability stain TAMRA (magenta) and inoculated onto a confluent HT29 monolayer at an m.o.i. of 100 : 1. We observed a 10-fold increase in both bacterial total cellular association (10–15 µg ml^−1^) and intracellular bacteria (10 µg ml^−1^) by gentamicin protection assay at 3 hpi [Fig. 1b(i, ii)]. The induced bacteria did not show a change in autoagglutination after 3 h, suggesting the invasive phenotype was not due to increased clumping [Fig. 1c(i)]. A novobiocin-induced increase in autoagglutination at 24 h was observed [Fig. 1c(i)]. However, the majority of *C. jejuni* in all conditions had agglutinated at this timepoint. Novobiocin (10 µg ml^−1^) treatment was chosen as the standard dose for subsequent infection assays, denoted as ‘*C. jejuni* (SI)’. *C. jejuni* (SI) was associated with increased localization of bacterial clumps with integrin effector paxillin (Fig. 1d, green) early in infection (1 hpi). In addition, *C. jejuni* (SI) also preferentially localized to cell-to-cell junctions, suggesting penetrance into the junction interface (Fig. 1e). Interestingly, this was also associated with localized loss of *β*-catenin signal (Fig. 1f, green) associated with AJs.

### Induced invasive phenotype enhances kinetics of TJ disruption

Driven by the observation that the ‘*C. jejuni* SI’ phenotype promoted *C. jejuni*-mediated disruption of a component of AJs and promoted paracellular localization, an HT29-polarized monolayer model was used to investigate the ability of the *C. jejuni* (SI) invasive phenotype to invade less permissive differentiated epithelium. HT29 cells were grown for 30 days on transwell inserts before infection with TAMRA-stained untreated *C. jejuni*, or *C. jejuni* (SI), for 6 and 24 h. Polarization of the HT29 monolayer was evident by TJ formation and visualized by immunofluorescent labelling of occludin, a key structural component of the TJ assembly [Fig. 2a(i)]. There appeared to be moderate disruption of TJs when the cells were infected with *C. jejuni* for 6h [Fig. 2a(ii)]. However, *C. jejuni* (SI) appeared to cause total disruption of TJs and total penetrance into the monolayer after 24-h infection [Fig. 2a(iii)]. This was associated with a profound increase in the total association of viable bacteria for *C. jejuni* (SI) versus *C. jejuni* [Fig. 2a(iv)]. Single-cell quantification of cellular occludin intensity showed that untreated *C. jejuni* preferentially localized to cells with strong TJ signal at 6 h, with this trend being maintained at the 24-h timepoint [Fig. 2b(i)]. Conversely, cells with *C. jejuni* (SI) associated presented with a modest but significant loss in occludin intensity at 6 h with a total loss of TJ morphology and occludin intensity by 24-h infection associated with deep invasion of the viable *C. jejuni* (SI) into the formerly polarized monolayer [Fig. 2b(ii)].

### Induced invasive phenotype promotes morphological disruption of intestinal spheroids

To investigate further the invasive phenotype of *C. jejuni* (SI) affecting cell-cell junctions and cytoskeleton homeostasis in a more physiologically relevant model, a high-content screening infection approach using a 3D cell culture model was carried out by adapting a matrix-based overlay method which consisted of seeding HT29 cells grown as intestinal spheroids on Matrigel coated optically clear multiwell plates. HT29 cells seeded for 4 days in 4 mg ml^−1^ Matrigel, and the resulting HT29 spheroids were infected with viability-stained *C. jejuni*, *C. jejuni* (SI) and *S*. Typhimurium for 6 and 24 h and immunostained for actin, *β*-catenin and occludin to measure spheroid morphology, cytoskeletal remodelling and AJ and TJ disruption during infection. The uninfected intestinal spheroids possessed ideal apical/basolateral polarization as demonstrated by TJ polarization to the extracellular matrix facing external membranes of the spheroids [Fig. 3a(i)]. This correct orientation provides a physiological barrier as would be faced by *C. jejuni* during *in vivo* infection of the human intestine. An automated image analysis pipeline was developed to quantify spheroid morphometrics, bacteria association with spheroids, occludin, *β*-catenin and actin fluorescence signal within spheroids (Fig. 3b). For each condition, images were acquired at 25 sub-positions, with 50 Z-slices for each sub-position. In total, 250–300 spheroids were analysed per condition, with the entire spheroid Z-dimension quantified using maximum Z-projections maintaining Z-colocalization identity. *C. jejuni* (SI) demonstrated significantly more total association with spheroids as well as bacteria penetrance into the spheroid structure at 6 hpi [Fig. 3c(i, ii)]. This was not associated with significant disruption of spheroid morphology [Fig. 3c(iii, iv)], which was evident in the *S*. Typhimurium-positive control for spheroid disruption. This increase in viable bacteria was lost at 24 hpi [Fig. 3d(i, ii)]. Neither *C. jejuni* or *C. jejuni* (SI) demonstrated significant disruption of spheroid integrity at 24 hpi, with total loss of spheroid integrity seen with *S*. Typhimurium promoting cell death and disaggregation of spheroid structure.

### Induced invasive phenotype disrupts TJs during early infection and AJ in later infection

*C. jejuni* (SI) had displayed an increase in the kinetics to degrade TJs of polarized HT29 monolayers and *β*-catenin intensity in monolayers. Therefore, we investigated if cytoskeletal remodelling and TJ and AJ disruption were occurring in intestinal spheroids. Single-spheroid analysis of actin intensity showed that the actin cytoskeleton was not disrupted at 6 hpi. However, at 24 hpi, there was a significant loss of F-actin for *C. jejuni* (SI) and *S*. Typhimurium but not for non-induced *C. jejuni* [Fig. 4a(i, ii)]. There was a modest loss of TJ signal at 6 hpi for non-induced *C. jejuni* [Fig. 4b(i, ii)]; however, induction of the invasive secretory phenotype led to a striking total disruption of TJs, similar to that observed by *S*. Typhimurium. By 24 hpi, non-induced *C. jejuni* disrupted TJs, however to a lesser extent than induced invasive phenotype despite similar levels of invading bacteria. At 6 hpi, there was a modest increase in *β*-catenin intensity for *C. jejuni* and *C. jejuni* (SI) [Fig. 4c(i, ii)], possibly due to the remodelling of *β*-catenin to the sites of bacterial adherence. SL1344 was however capable of disrupting the AJs after 4-h infection. Both *C. jejuni* and * C. jejuni* (SI) were capable of degradation of *β*-catenin signal after 24-h infection, with *C. jejuni* (SI) displaying far greater disruption of AJs [Fig. 4c(i, ii)].

### Induced invasive phenotype triggered disruption of TJs and AJs require microtubule dynamics

*C. jejuni* (SI) was shown to profoundly increase the kinetics of TJ disruption. This phenotype was also associated with a unique and profound disruption of F-actin and total disruption of *β*-catenin labelled AJs by 24 hpi. To determine whether bacterial factor-mediated remodelling of actin and microtubule cytoskeleton contributed to this disruption of host homeostasis, *C. jejuni* (SI) was incubated with HT29 spheroids, which had been treated with either 33 µM nocodazole which

binds *β*-tubulin, preventing polymerization of microtubules, and enhances GTPase activity [36] 3 h (maintained at 3 µM during infection), or 10 µM cytochalasin D which caps the barbed end of filamentous actin, disrupting the polymerization-depolymerization homeostasis, resulting in depolymerization of F-actin [36] for 90 min (maintained at 1 µM during infection) to inhibit cytoskeletal remodelling. Actin cytoskeleton disruption but not microtubule disruption enhanced bacterial total association [Fig. 5a(i)]. Both actin and microtubule remodelling disruption caused loss of occludin signal, with nocodazole affecting it to a lesser extent. Interestingly, nocodazole treatment but not cytochalasin D treatment reduced the ability of *C. jejuni* (SI) to disrupt TJs [Fig. 5a(ii), b(i)]. At 24 hpi, both inhibitors led to total disruption of the occludin signal [Fig. 5b(ii)]. Interestingly, cytochalasin D reduced the *β*-catenin signal from baseline at 6 hpi [Fig. 5c(i)]; however, treatment with nocodazole totally abrogated the ability of the *C. jejuni* (SI) to disrupt the *β*-catenin signal at 24 hpi, returning the *β*-catenin spheroid intensities to that of baseline *C. jejuni* infection [Fig. 5c(ii)], implicating microtubule remodelling as a key target of *C. jejuni*-mediated disruption of AJs.

## Discussion

Changes in DNA supercoiling topology in response to external stimuli during infection allow for bacterial pathogens to take advantage of supercoiling-sensitive promoters to regulate virulence and stress response gene expression. This provides the tools to adapt to changing environments as well as preparing for virulence. In *C. jejuni*, the relaxation of negative supercoiling is associated with key factors present uniquely in the human intestine, such as bile salt sodium deoxycholate [37,38]. This study modelled this relaxed state to link DNA supercoiling-mediated global regulation of *C. jejuni* virulence gene expression to the degradation and remodelling of host cell mechanisms, as well as paracellular translocation using *in vitro* models of infection of the human epithelium.

Here, we visualized 2D and 3D intestinal epithelium models challenged with *C. jejuni* and the same strain in which the relaxation of DNA supercoiling was pharmacologically induced by subinhibitory levels of novobiocin [*C. jejuni* (SI)] to induce invasion. This approach allows for the elucidation of the cellular mechanisms being modulated during the switching from a less-invasive to virulent phenotype, a key question in the study of *C. jejuni* pathogenicity.

Baseline *C. jejuni* is visibly associated with HT29 intestinal epithelial cells in very low numbers. However, the relaxation of DNA supercoiling via preincubation with 10 µg ml^−1^ novobiocin resulted in the formation of large cell-cell border-associated clusters of adherent *C. jejuni* (SI), which were termed ‘invasion clusters’. *C. jejuni* (SI) also displayed a significant increase in both attachment and invasion of HT29 cells. Observation of these invasion clusters immediately raised the question: are the * C. jejuni* self-aggregating before contact with the epithelium? Surprisingly, no significant change in autoagglutination potential was observed for *C. jejuni* (SI), suggesting that the aggregation observed upon association with the HT29 epithelium was a hallmark of specific interaction with the host cells. Recruitment and phosphorylation of paxillin have been suggested as a key component of the adhesion-induced host cell signalling cascade involved in *C. jejuni* internalization [17]. During early-stage infection, * C. jejuni* (SI) could be observed clustering to paxillin-rich membrane structures when adhering to the HT29 cells. Surprisingly, at all timepoints, untreated *C. jejuni* were not observed to associate closely to either paxillin-positive plasma membrane or even to the cell island peripheries. These data suggested that the relaxation of DNA supercoiling promotes association and clustering of *C. jejuni* with focal adhesions, possibly recruiting paxillin to the site of adherence to the plasma membrane as well as promoting specific targeting to the focal complexes in an *in vitro* model in which Fn ECM component is absent. 2D invasion assays revealed that the most profound effect the relaxation of DNA SI phenotype had on the host cells was the disruption of TJ and AJs. Therefore, these host mechanisms were investigated using a less permissive, physiologically relevant 3D spheroid-based high-content invasion screen. Correctly polarized HT29 intestinal spheroids were infected with *C. jejuni*, *C. jejuni* (SI) and * S.* Typhimurium as a positive control for TJ and AJ disruption, for 6 and 24 h. Interestingly, significantly more viable *C. jejuni* (SI) is associated with spheroids early in infection, with confocal imaging showing invasion of bacteria into spheroids marked by localization at cell-cell junctions, which was also observed in monolayer cultures, while *C. jejuni* is predominantly associated with spheroid borders. This was associated with modest condensation of the spheroid area likely due to the disruption of cell-cell contacts and cytoskeletal organization.

As *C. jejuni* preferentially infects through the basolateral membrane, we proposed that the induced invasiveness of *C. jejuni* (SI) could be promoting the degradation of TJs and AJs to promote paracellular trafficking of the bacteria. *Escherichia coli*, *Salmonella enterica* and *Shigella flexneri* have all been shown to utilize T3SS system effectors to breach the AJ obstacle during paracellular translocation [5,39,41]. *β*-Catenin and *α*-catenin have been shown to be recruited to sites of *Listeria* adherence to cells and be required for cytoskeletal remodelling and invasion [42]. In *S*. Typhimurium, the SPI-I effector protein AvrA which plays a role in modulating immune response to the destructive effects of early-stage *Salmonella* SPI-I effectors, via stabilizing TJs and inhibiting NFκB signalling, also plays a role in dysregulation of AJ [43,44].

Firstly, we measured the AJ effector *β*-catenin during the infection of intestinal monolayers. What was immediately evident was that even *C. jejuni* in its less-invasive form seemed to preferentially localize to the cell-cell peripheries delineated by *β*-catenin signal. However, with *C. jejuni* (SI), individual bacteria were observed to be associating in the paracellular space, suggesting that these bacteria were not just sitting on the plasma membrane but entering the cell-cell junction. Surprisingly, the relaxation of DNA SI invasion clusters caused profound localized degradation of the fluorescent *β*-catenin signal. A massive loss of *β*-catenin signalling was observed during *C. jejuni* (SI) infection in HT29 intestinal spheroids at 24 hpi. Interestingly, pharmacological inhibition of actin polymerization partially rescued this loss; however, the inhibition of microtubule dynamics totally rescued this *C. jejuni*-induced phenotype, suggesting for the first time that components of this secretory phenotype may be directly targeting the degradation of *β*-catenin. This was similar to the observation that *Salmonella* SPI-I effector AvrA modulated *β*-catenin via promoting degradation and nuclear targeting, which in turn disrupts the maintenance of AJs [43,45]. The degradation of *β*-catenin, which also functions as a nuclear transcription activator, by invasive *C. jejuni* could have profound consequences on the modulation of host cell proliferation and immune response during infection.

*C. jejuni* (SI) was also found to have a profound effect on the organization of occludin-positive TJs of polarized HT29 monolayers. TJs are targets for degradation by a variety of invading gastrointestinal pathogens such as *E. coli* (occludin dissociation from TJ), *Vibrio cholera* (metalloprotease degradation of the cytosolic face of occludin) and *S*. Typhimurium during the invasion. This observation supports several studies which reported the protease-mediated degradation of TJs during infection, thought to be vital for paracellular translocation to the basolateral membrane of the intestinal epithelium [41,46]. After 6-h infection, *C. jejuni* (SI) had caused localized disruption of TJs and total disruption of occludin intensity by 24 hpi. Untreated *C. jejuni* had a minimal effect at 6 hpi but was capable of localized disruption of occludin signal after 24-h infection, while still maintaining low adherent numbers. Suggesting that the relaxation of DNA supercoiling primes *C. jejuni* (SI) for virulence, but incubation for extended periods of time in the presence of polarized HT29 cells promotes secretion of similar virulence factors at a lower rate. It was immediately apparent upon quantification of F-actin intensity within the spheroids that *C. jejuni* (SI) could remodel the actin cytoskeleton after 6-h infection. It is important to note that the decrease in F-actin signal may be attributed to the redistribution of the actin signal to specific subcellular localities, or it may be due to direct depolymerization of F-actin by the bacteria. A similar profile of occludin signal degradation was observed in HT29 intestinal spheroids as was observed using the polarized monolayer model. This provided further evidence to the hypothesis that the relaxation of DNA supercoiling is priming *C. jejuni* for TJ disruption and that environmental conditions result in a driving of the population towards this phenotype upon prolonged exposure to the host cell environment. TJ formation is dependent upon the actin cytoskeleton; therefore, total disruption of occludin signal was observed during cytochalasin D inhibition at 24 h. However, inhibition of microtubule dynamics with nocodazole partially rescued *C. jejuni* (SI)-mediated degradation of TJs during early infection.

In summary, this study has, for the first time, directly correlated a previously described inducible, DNA supercoiling-regulated invasive phenotype in *C. jejuni* with the modification of host epithelial cellular mechanisms. Specifically, this study reveals that this more invasive phenotype is driven through the disruption of TJs, implying a possible paracellular route of invasion.

## Supplementary material

10.1099/mic.0.001560 Uncited Supplementary Material 1.

## Abbreviations

AJ: adherens junction
hpi: hours post-infection
MH: Mueller–Hinton
RT: room temperature
SI: supercoiling induced
TJ: tight junction
