# Supplementary material for: An invasive phenotype induced by relaxation of DNA supercoiling in Campylobacter jejuni triggers disruption of tight junctions of intestinal spheroids
Source: Microbiology (Reading). 2025 Jun 5;171(6):001560. doi: 10.1099/mic.0.001560 (PMC12141748; doi:10.1099/mic.0.001560)
Supplement: Uncited Supplementary Material 1. [file mic-171-01560-s001.pdf]

Supplementary Information

Cellprofiler analysis pipeline

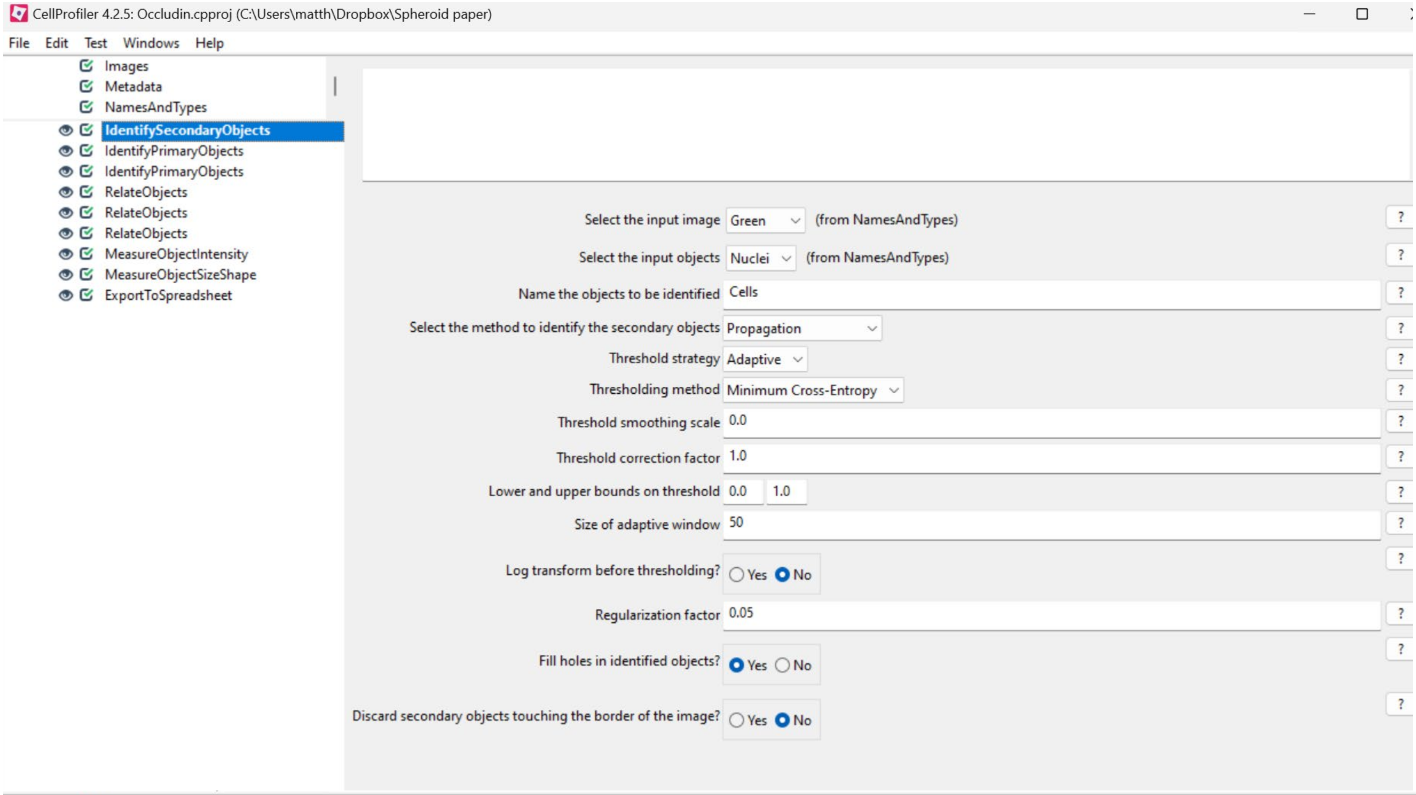

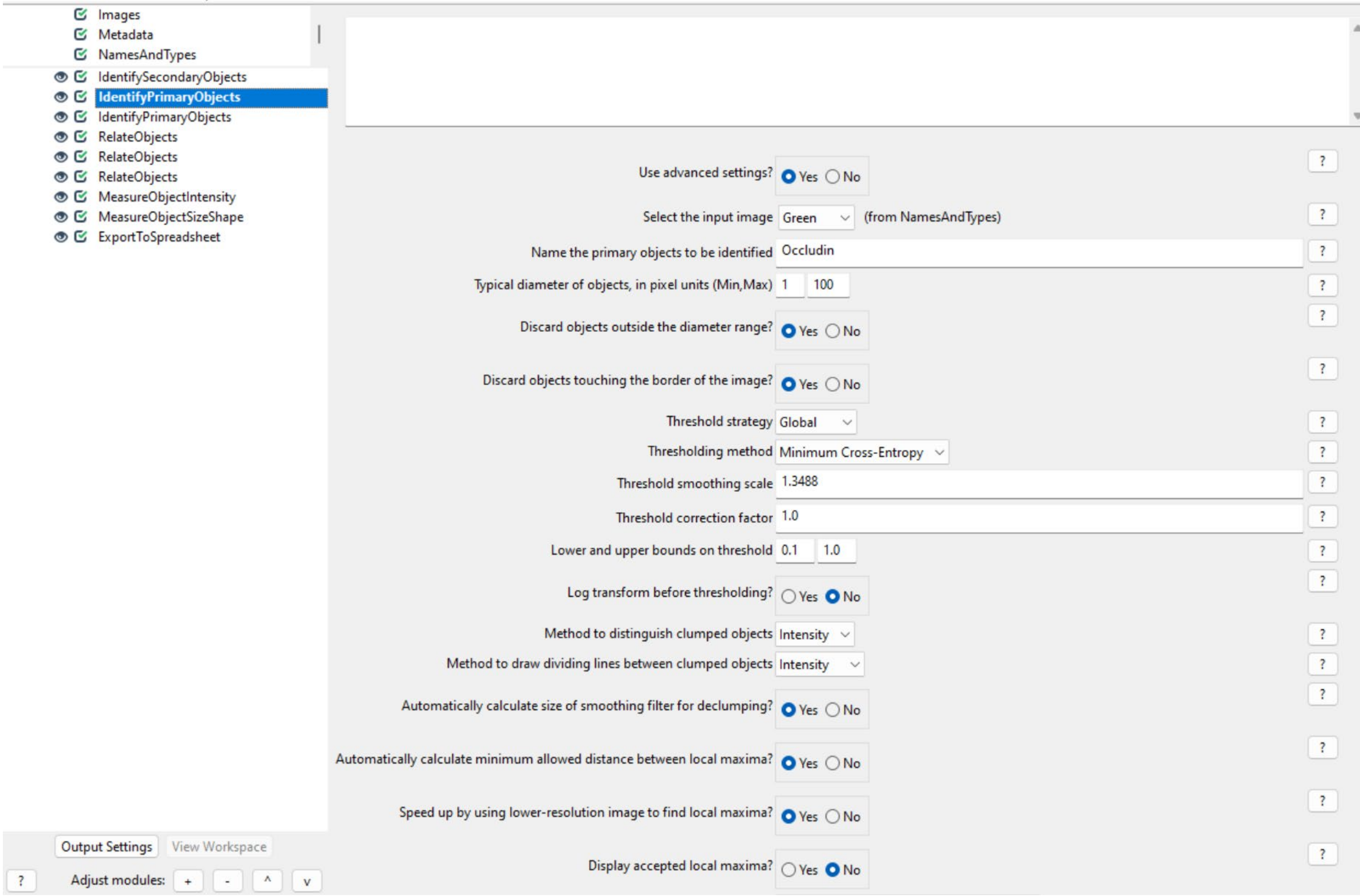

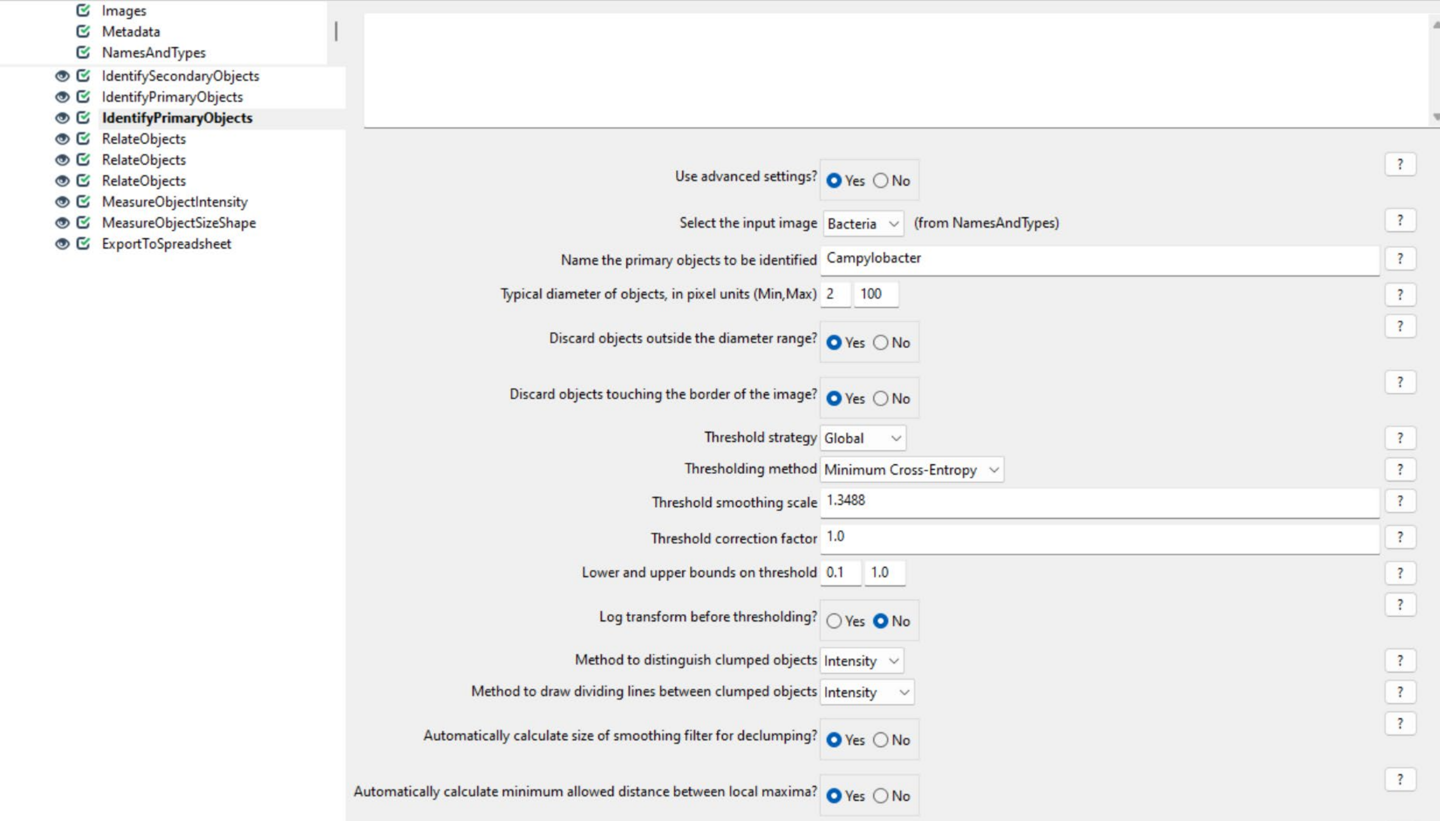
Images
Metadata
NamesAndTypes
IdentifySecondaryObjects
IdentifyPrimaryObjects
IdentifyPrimaryObjects
RelateObjects
RelateObjects
RelateObjects
MeasureObjectIntensity
MeasureObjectSizeShape
ExportToSpreadsheet
Use advanced settings? Yes No
Select the input image Bacteria (from NamesAndTypes)
Name the primary objects to be identified Campylobacter
Typical diameter of objects, in pixel units (Min,Max) 2 100
Discard objects outside the diameter range? Yes No
Discard objects touching the border of the image? Yes No
Threshold strategy Global
Thresholding method Minimum Cross-Entropy
Threshold smoothing scale 1.3488
Threshold correction factor 1.0
Lower and upper bounds on threshold 0.1 1.0
Log transform before thresholding? Yes No
Method to distinguish clumped objects Intensity
Method to draw dividing lines between clumped objects Intensity
Automatically calculate size of smoothing filter for declumping? Yes No
Automatically calculate minimum allowed distance between local maxima? Yes No
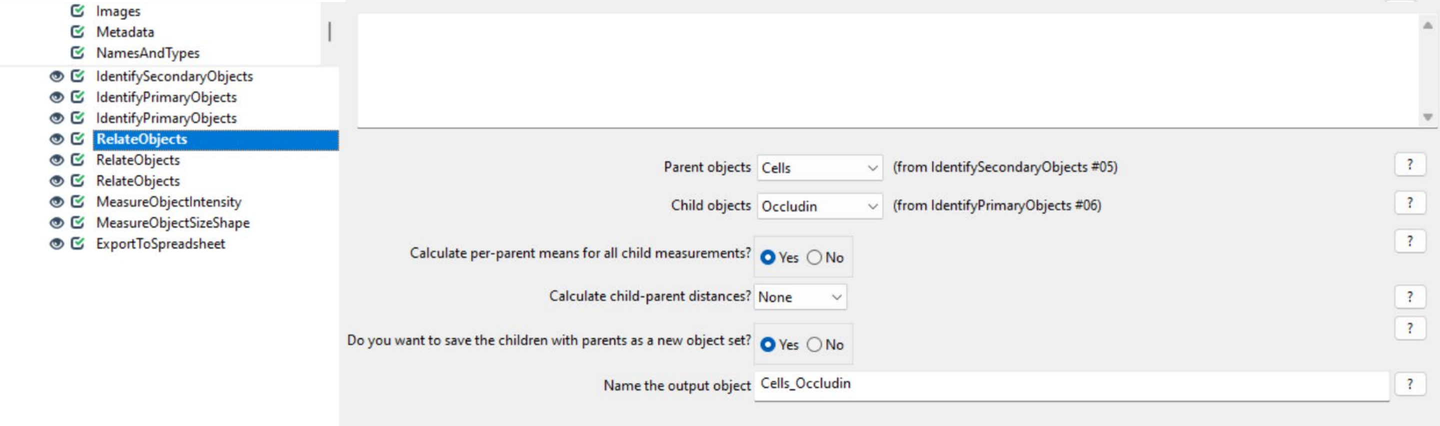
Images
Metadata
NamesAndTypes
IdentifySecondaryObjects
IdentifyPrimaryObjects
IdentifyPrimaryObjects
RelateObjects
RelateObjects
RelateObjects
MeasureObjectIntensity
MeasureObjectSizeShape
ExportToSpreadsheet
Parent objects Cells (from IdentifySecondaryObjects #05)
Child objects Occludin (from IdentifyPrimaryObjects #06)
Calculate per-parent means for all child measurements? Yes No
Calculate child-parent distances? None
Do you want to save the children with parents as a new object set? Yes No
Name the output object Cells_Occludin
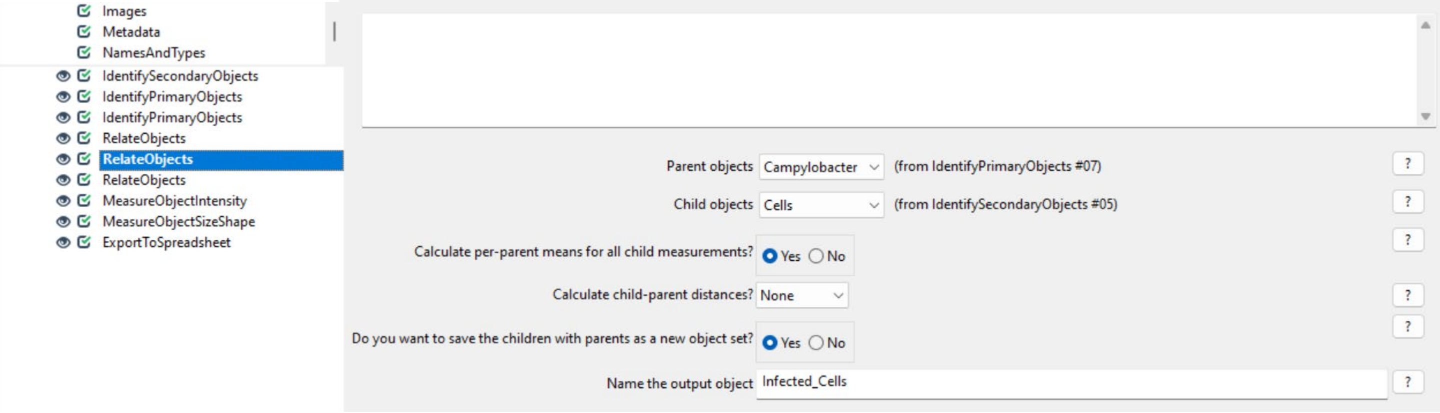
Images
Metadata
NamesAndTypes
IdentifySecondaryObjects
IdentifyPrimaryObjects
IdentifyPrimaryObjects
RelateObjects
RelateObjects
RelateObjects
MeasureObjectIntensity
MeasureObjectSizeShape
ExportToSpreadsheet
Parent objects Campylobacter (from IdentifyPrimaryObjects #07)
Child objects Cells (from IdentifySecondaryObjects #05)
Calculate per-parent means for all child measurements? Yes No
Calculate child-parent distances? None
Do you want to save the children with parents as a new object set? Yes No
Name the output object Infected_Cells
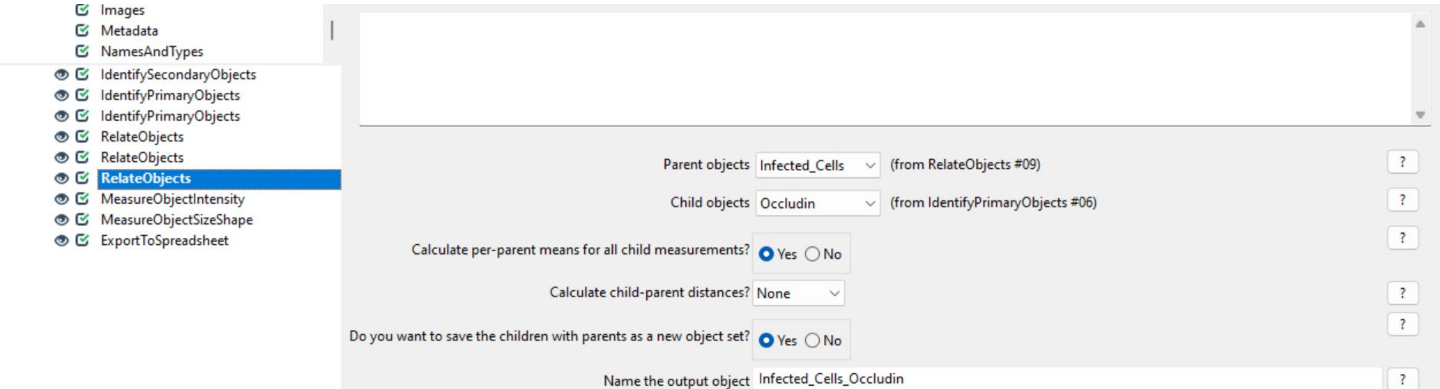
Images
Metadata
NamesAndTypes
IdentifySecondaryObjects
IdentifyPrimaryObjects
IdentifyPrimaryObjects
RelateObjects
RelateObjects
RelateObjects
MeasureObjectIntensity
MeasureObjectSizeShape
ExportToSpreadsheet
Parent objects Infected_Cells (from RelateObjects #09)
Child objects Occludin (from IdentifyPrimaryObjects #06)
Calculate per-parent means for all child measurements? Yes No
Calculate child-parent distances? None
Do you want to save the children with parents as a new object set? Yes No
Name the output object Infected_Cells_Occludin

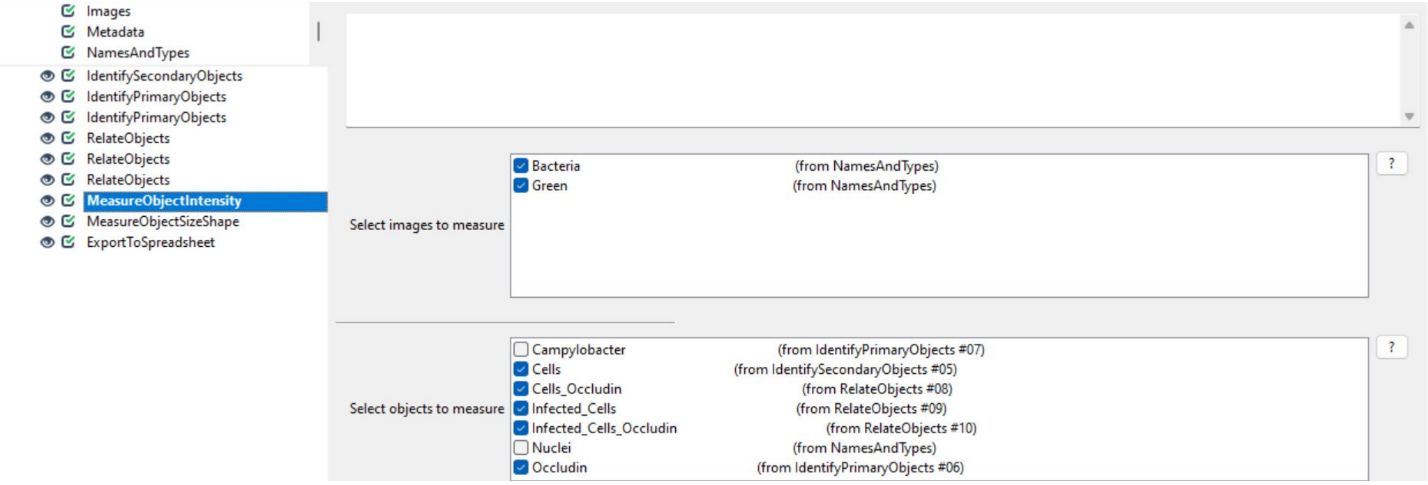
Images
Metadata
NamesAndTypes
IdentifySecondaryObjects
IdentifyPrimaryObjects
IdentifyPrimaryObjects
RelateObjects
RelateObjects
RelateObjects
MeasureObjectIntensity
MeasureObjectSizeShape
ExportToSpreadsheet
Select images to measure
Bacteria (from NamesAndTypes)
Green (from NamesAndTypes)
Select objects to measure
Campylobacter (from IdentifyPrimaryObjects #07)
Cells (from IdentifySecondaryObjects #05)
Cells_Occludin (from RelateObjects #08)
Infected_Cells (from RelateObjects #09)
Infected_Cells_Occludin (from RelateObjects #10)
Nuclei (from NamesAndTypes)
Occludin (from IdentifyPrimaryObjects #06)

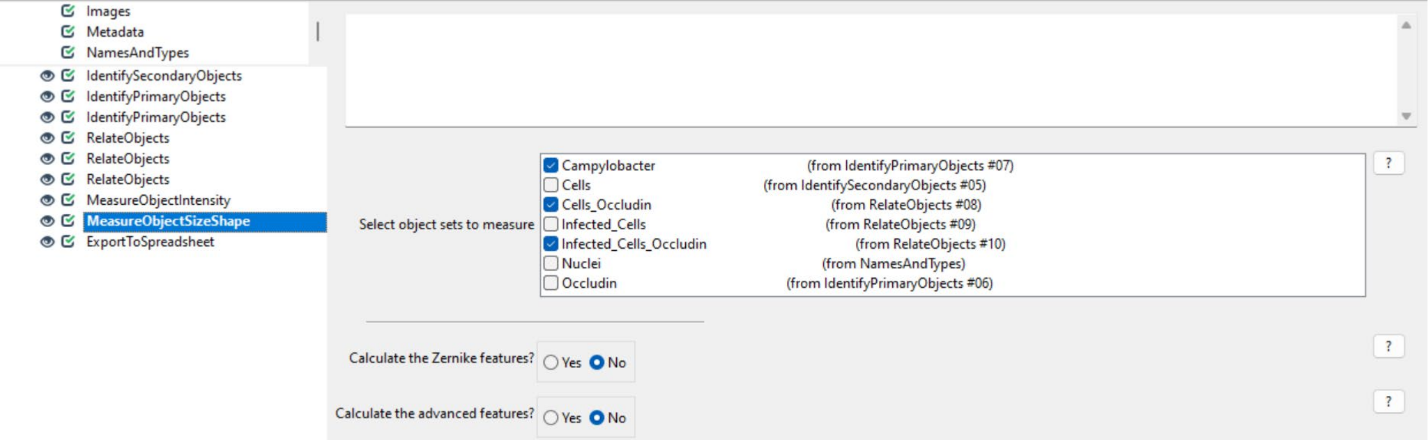
Images
Metadata
NamesAndTypes
IdentifySecondaryObjects
IdentifyPrimaryObjects
IdentifyPrimaryObjects
RelateObjects
RelateObjects
RelateObjects
MeasureObjectIntensity
MeasureObjectSizeShape
ExportToSpreadsheet
Select object sets to measure
Campylobacter (from IdentifyPrimaryObjects #07)
Cells (from IdentifySecondaryObjects #05)
Cells_Occludin (from RelateObjects #08)
Infected_Cells (from RelateObjects #09)
Infected_Cells_Occludin (from RelateObjects #10)
Nuclei (from NamesAndTypes)
Occludin (from IdentifyPrimaryObjects #06)
Calculate the Zernike features? Yes No
Calculate the advanced features? Yes No

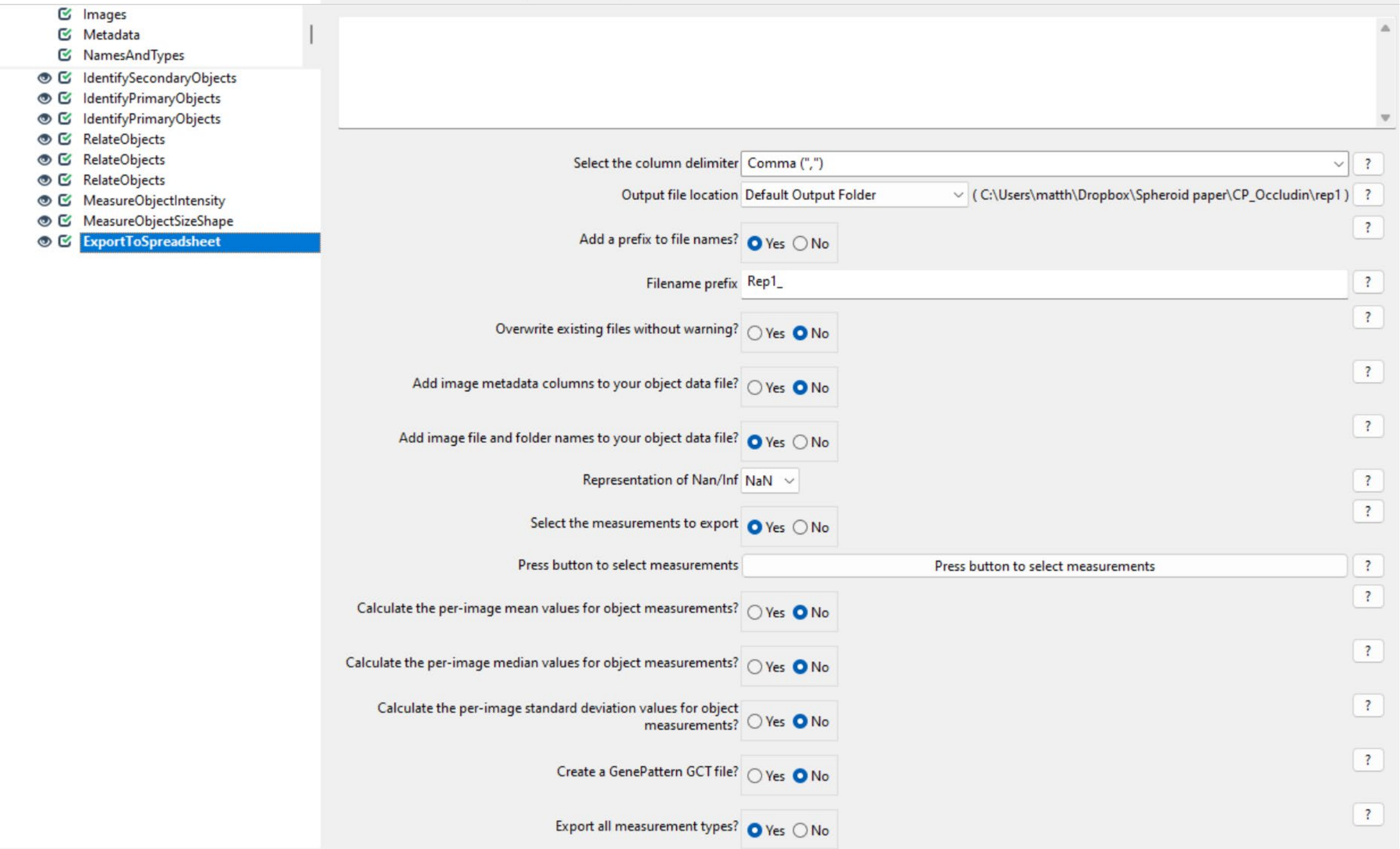
Images
Metadata
NamesAndTypes
IdentifySecondaryObjects
IdentifyPrimaryObjects
IdentifyPrimaryObjects
RelateObjects
RelateObjects
RelateObjects
MeasureObjectIntensity
MeasureObjectSizeShape
ExportToSpreadsheet
Select the column delimiter Comma (",")
Output file location Default Output Folder (C:\Users\matth\Dropbox\Spheroid paper\CP_Occludin\rep1)
Add a prefix to file names? Yes No
Filename prefix Rep1_
Overwrite existing files without warning? Yes No
Add image metadata columns to your object data file? Yes No
Add image file and folder names to your object data file? Yes No
Representation of Nan/Inf NaN
Select the measurements to export Yes No
Press button to select measurements Press button to select measurements
Calculate the per-image mean values for object measurements? Yes No
Calculate the per-image median values for object measurements? Yes No
Calculate the per-image standard deviation values for object measurements? Yes No
Create a GenePattern GCT file? Yes No
Export all measurement types? Yes No

Columbus analysis pipeline

Analysis Sequence "Mothra_Initial"

Step	Input	Method	Output
**Input Image**	**Flatfield Correction :** Basic Brightfield Correction<br>**Stack Processing :** Maximum Projection<br>**Min. Global Binning :** Dynamic		
**Find Image Region**	**Channel :** Alexa 568<br>**ROI :** None	**Method :** Common Threshold<br>Threshold : 0.1<br>Split into Objects<br>Area : > 500 μm^2<br>Fill Holes	Output Population : Spheroid<br>Output Region : Spheroid
**Find Nuclei**	**Channel :** HOECHST 33342 - extended<br>**ROI :** Spheroid<br>**ROI Region :** Spheroid	**Method :** C<br>Common Threshold : 0.4<br>Area : > 30 μm^2<br>Splitting Coefficient : 7.0<br>Individual Threshold : 0.65<br>Contrast : > 0.05	Output Population : Nuclei
**Find Cytoplasm**	**Channel :** Alexa 568<br>**Nuclei :** Nuclei	**Method :** A<br>Individual Threshold : 0.15<br>Restrictive Region : Spheroid	
**Find Spots**	**Channel :** Alexa 488<br>**ROI :** Spheroid<br>**ROI Region :** Spheroid	**Method :** B<br>Detection Sensitivity : 0.1<br>Splitting Sensitivity : 0.5<br>Calculate Spot Properties	Output Population : Bacteria
**Calculate Intensity Properties (2)**	**Channel :** Alexa 568<br>**Population :** Spheroid<br>**Region :** Spheroid	**Method :** Standard Mean	Property Prefix : Intensity Spheroid B-Catenin
**Calculate Intensity Properties (3)**	**Channel :** Alexa 647	**Method :** Standard	Property Prefix :

	**Population :** Spheroid<br>**Region :** Spheroid	Mean	Intensity Spheroid Phalloidin-647
**Calculate Intensity Properties**	**Input**	**Method**	**Output**
	**Channel :** Alexa 488<br>**Population :** Spheroid<br>**Region :** Spheroid	**Method :** Standard<br>Mean	Property Prefix :<br>Intensity Cell alexa-488 Bacteria
**Calculate Morphology Properties**	**Input**	**Method**	**Output**
	**Population :** Nuclei<br>**Region :** Nucleus	**Method :** Standard<br>Area<br>Roundness	Property Prefix :<br>Nucleus
**Calculate Morphology Properties (2)**	**Input**	**Method**	**Output**
	**Population :** Spheroid<br>**Region :** Spheroid	**Method :** Standard<br>Area<br>Roundness	Property Prefix :<br>Spheroid
**Define Results**	**Results**		
	**Method :** List of Outputs<br>**Population : Bacteria**<br>Apply to All : Mean<br>Relative Spot Intensity : Mean<br>Corrected Spot Intensity : Mean<br>Uncorrected Spot Peak Intensity : Mean<br>Spot Contrast : Mean<br>Spot Background Intensity : Mean<br>Spot Area [px²] : Mean<br>Region Intensity : Mean<br>Spot to Region Intensity : Mean<br><br>**Population : Nuclei**<br>Number of Objects<br>Apply to All : Mean<br>Nucleus Area [µm²] : Mean<br>Nucleus Roundness : Mean<br><br>**Population : Spheroid**<br>Number of Objects<br>Apply to All : Mean<br>Total Spot Area : Mean<br>Relative Spot Intensity : Mean<br>Number of Spots : Mean<br>Number of Spots per Area of Spheroid : Mean<br>Intensity Spheroid B-Catenin Mean : Mean<br>Intensity Spheroid Phalloidin-647 Mean : Mean<br>Intensity Cell alexa-488 Bacteria Mean : Mean<br>Spheroid Area [µm²] : Mean<br>Spheroid Roundness : Mean		

Supplementary Table 1. Image analysis pipeline

Blank | Blank +novobiocin 24h

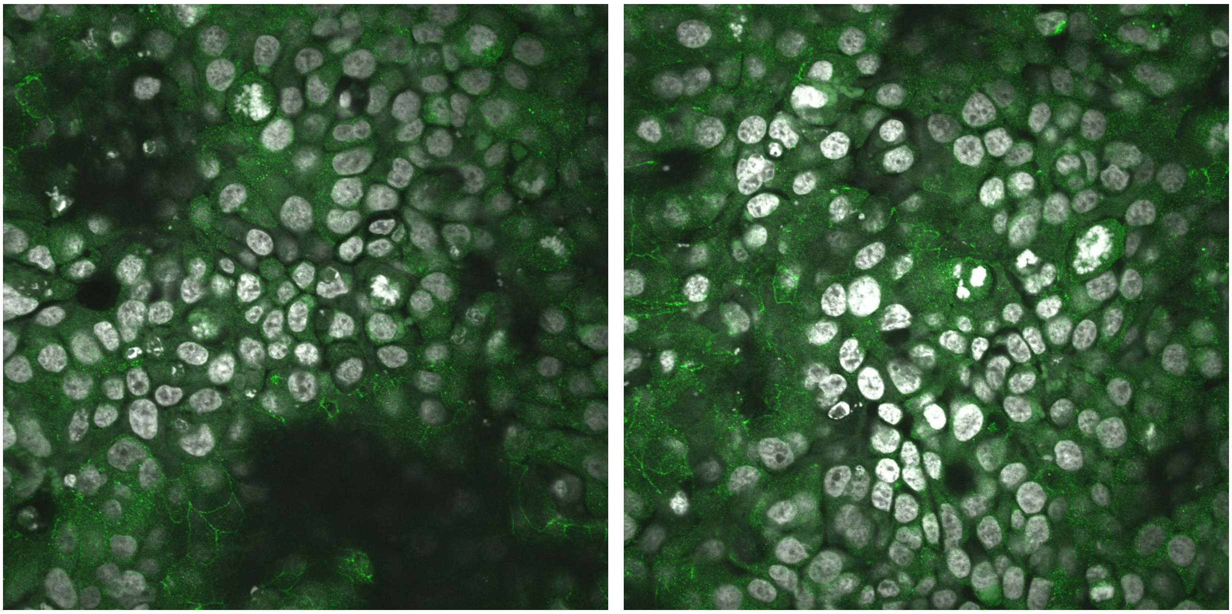

Supplementary Fig 1. Apical confocal Z-slice of HT29 polarised monolayer in controlled media and controlled media +10ug/ml novobiocin for 24h. Novobiocin did not affect the present of tight junctions.
